# Multibounce and Subsurface Scattering of H Atoms Colliding with a van der Waals Solid

**DOI:** 10.1021/acs.jpca.1c03433

**Published:** 2021-06-28

**Authors:** Nils Hertl, Alexander Kandratsenka, Oliver Bünermann, Alec M. Wodtke

**Affiliations:** † Institut für physikalische Chemie, Universität Göttingen, Tammannstrasse 6, 37077 Göttingen, Germany; ‡ Department of Dynamics at Surfaces, Max-Planck Institute for Biophysical Chemistry, am Faßberg 11, 37077 Göttingen, Germany; § International Center for Advanced Studies of Energy Conversion, Georg-August University of Göttingen, Tammannstrasse 6, 37077 Göttingen, Germany

## Abstract

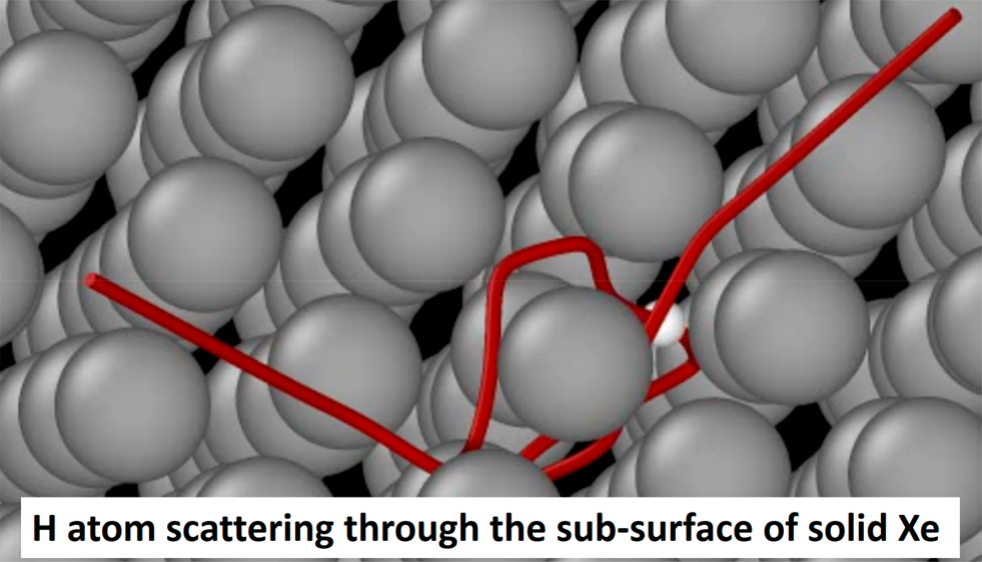

We report the results of inelastic differential scattering experiments and full-dimensional molecular dynamics trajectory simulations for 2.76 eV H atoms colliding at a surface of solid xenon. The interaction potential is based on an effective medium theory (EMT) fit to density functional theory (DFT) energies. The translational energy-loss distributions derived from experiment and theory are in excellent agreement. By analyzing trajectories, we find that only a minority of the scattering results from simple single-bounce dynamics. The majority comes from multibounce collisions including subsurface scattering where the H atoms penetrate below the first layer of Xe atoms and subsequently re-emerge to the gas phase. This behavior leads to observable energy-losses as large as 0.5 eV, much larger than a prediction of the binary collision model (0.082 eV), which is often used to estimate the highest possible energy-loss in direct inelastic surface scattering. The sticking probability computed with the EMT-PES (0.15) is dramatically reduced (5 × 10^–6^) if we employ a full-dimensional potential energy surface (PES) based on Lennard-Jones (LJ) pairwise interactions. Although the LJ-PES accurately describes the interactions near the H–Xe and Xe–Xe energy minima, it drastically overestimates the effective size of the Xe atom seen by the colliding H atom at incidence energies above about 0.1 eV.

## 1 Introduction

Collisions of atoms and molecules with surfaces typically lead to two experimentally identifiable outcomes: direct inelastic scattering (DIS) and trapping followed by thermal desorption (TD).^1^ DIS may exhibit maximum flux near the specular scattering angle similar to reflection of light from a flat-mirrored surface. Such behavior is often described as “single-bounce” scattering^2,3^ since measured translational inelasticity is typically consistent with simple models where momentum is exchanged between the projectile and a single surface atom.^4,5^ Furthermore, the measured translational^6^ and internal^7^ energy distributions of scattered particles are nonthermal.^8^ By contrast, TD occurs when the energy lost in the initial collision is sufficient to prevent the projectile’s escape from the surface.^9^ Here, a sequence of many collisions brings the projectile to thermal equilibrium with the surface.^10^ This may also involve surface penetration followed by resurfacing.^11,12^

Scattering of Ne, Ar, and Xe from liquid molecular surfaces^13^ as well as Ne^14−16^ scattering from *n*-hexylthiolate self-assembled monolayer (SAM) on Au(111) and water ice^17^ also show DIS as well as TD scattering. However, here the thermal component may hide more complex dynamics.^14−18^ Classical trajectories showed that a Boltzmann component could arise even when the interaction times are on the picosecond time scale and where trajectories involve only a single inner turning point during the Ne-SAM collision.^15^ This has been attributed to the excitation of the polyatomic surface to high-energy conformers and rapid intramolecular vibrational redistribution through anharmonic intramolecular coupling, allowing many degrees of freedom of the surface to be coupled to the rare gas atom’s motion.^16^ Hyperthermal Xe scattering experiments from SAM even showed three velocity components: DIS, TD, and a direct scattering process, dubbed “channel-directed ejection”, where hyperthermal Xe penetrates the channels in the SAM before experiencing a repulsive interaction resulting in nonthermalized ejection.^18^

There have been no such observations of complex scattering dynamics involving multiple bounces or subsurface penetration from simple (atomic) surfaces. Molecular dynamics (MD) simulations of Ar colliding with Pt(111) provide evidence suggesting that DIS may be unlikely if more than one bounce occurs.^19^ Electronically nonadiabatic MD simulations of H scattering from fcc metal(111) surfaces^20^ suggest that penetration to the subsurface leads exclusively to trapping, as electronic friction experienced by the H atom is quite strong in the subsurface.^11,12^ These trajectory simulations involved multibounce trajectories, but because there is no definitive theory for nonadiabatic dynamics, we cannot be certain that multibounce dynamics are accurately represented.^21^ Despite this, there appears to us no reason why multibounce or subsurface DIS should not be demonstrably observable.

Experimental detection of multibounce and subsurface scattering is difficult. Distinguishing DIS from TD exploits the fact that the measured speed distributions of scattered and desorbing particles often produce two peaks,^22^ one with high velocities where a relatively small fraction of the incidence energy is lost to the surface (DIS) and one reflecting the low speeds of particles that have reached thermal equilibrium with the surface (TD). By using hyperthermal beams and low surface temperatures, the measured speeds of particles undergoing DIS can be resolved from those undergoing TD. It appears likely that the energy losses associated with multibounce, subsurface, and single-bounce DIS overlap with one another and therefore may require special conditions and methods to be detected.

In this work, we present results from inelastic scattering experiments involving H atom collisions with surfaces of solid xenon. We employ a nearly monoenergetic beam of H atoms with incidence translational energy *E*_i_ of 2.76 eV. The H atom beam is incident at ϑ_i_ = 45° from the surface normal, and scattered H atoms are detected at an angle ϑ_s_ = 45° from the surface normal. These conditions strongly favor observation of DIS. The energy-loss distribution exhibits a maximum at the energy predicted by a binary line-of-centers (LOC) collision model, suggesting the importance of single-bounce dynamics. In addition to this feature, a second feature exhibiting much larger energy-loss is seen. We simulate the scattering using classical MD simulations with a full dimensional potential energy surface (PES)^12,23^ fitted to density function theory (DFT) data; the simulation is in excellent agreement with experiment and is used to investigate the dynamical processes giving rise to the energy-loss spectrum. This shows that multibounce DIS including subsurface scattering makes up the majority of events seen in the experimentally derived energy-loss distribution.

## 2 Methods

The H atom scattering apparatus has been described elsewhere,^24^ and a review has recently appeared.^25^ Briefly, H atoms were generated by photodissociation of a supersonic molecular beam of hydrogen iodide with pulses of laser light at 212.5 nm, producing H atoms with incidence energy *E*_i_ = 2.76 eV and an energy uncertainty δ*E*_i_ ∼ 0.005 eV. H atoms traveling normal to the molecular beam scatter from the Xe sample that was condensed on a Au(111) substrate held at 45 K by cold He gas. Scattered H atoms were excited to a long-lived Rydberg state by two laser pulses, one exciting the 1s → 2p transition at 121.6 nm and another the 2p → 34d transition at 365.9 nm. The resulting metastable atoms travel 25 cm without radiative loss and are field-ionized and detected by a multichannel plate detector. A multichannel scaler records the arrival time and the calibrated flight length is used to obtain H atom speeds.

We performed classical MD trajectory calculations using a full-dimensional potential energy surface (PES) obtained by fitting an effective medium theory (EMT) function to DFT data. This procedure followed our previous work using PESs for H interacting with metals.^12,20,23^ The DFT input data was generated using VASP 5.3.5^26−29^ with the PBE functional^30^ and D2 van der Waals corrections usingGrimme’s method.^31^ Xe was modeled as a 2 × 2 fcc (111) slab with 4 layers. The Brillouin zone was sampled with a 4 × 4 × 1 gamma-centered *k*-point mesh, using the sampling scheme of Monkhorst and Pack.^32^ The plane wave cutoff energy was set to 250 eV. The interaction between the valence and core electrons have been described by the projector augmented wave approach.^33^ The optimum lattice constant of an ideal Xe crystal has been found to be 6.065 Å. To avoid interactions between the Xe slab and its periodic images in the *z*-direction, a vertical distance between unit cells of 13 Å has been applied in the *z*-direction. For the MD simulations with our EMT-PES, we modeled Xe as a (6 × 6) 6-layered slab with periodic boundary conditions. The EMT parameters resulting from the fit to the DFT data are presented in Table 1.

**Table 1:** Parameters Needed to Construct the H on Solid Xe Full-Dimensional Effective Medium Theory Potential

EMT-based potential fit to DFT data							
	η_2_/Å^–1^	*n*_0_/Å^–3^	*E*_0_/eV	λ/Å^–1^	*V*_0_/eV^–1^	κ/Å^–1^	*s*_0_/Å^–1^
H	0.838	0.193	–0.743	2.530	0.638	1.641	0.741
Xe	2.181	0.056	–0.160	1.765	0.042	2.499	2.370

We also constructed a Lennard-Jones (LJ) pair potential PES in full dimensions. The parameters used in that potential are shown in Table 2. The LJ parameters for the H–Xe interaction were obtained from the analysis of H–Xe scattering experiments and are the best available.^34^

**Table 2:** Parameters Needed to Construct the H on Solid Xe Full-Dimensional LJ Potential

LJ potential		
	σ/Å	ε/eV
H–Xe^34^	3.935	0.020
Xe–Xe^35^	3.98	0.019

The thermal motion of the Xe atoms was explicitly treated in the MD simulations; the Xe atom’s initial positions and velocities were sampled from equilibrium simulations at 45 K with the deepest layer held fixed. In each trajectory, the H atom was placed 6 Å above the surface with random lateral positions. The initial conditions were chosen so that they agree with the experiment. We launched 10^6^ trajectories to get a reasonable amount of scattering events that meet the experimental scattering conditions. The H atom is considered to be scattered, when its final vertical position is again 6.05 Å above the surface. The MD simulations were performed in an *NVE* ensemble with an integration time step of 0.1 fs. The PESs and propagation algorithms used in this work are implemented into the md_tian 2 package, written in Fortran and publicly available.^36^

## 3 Results and Discussion

**Figure 1:**
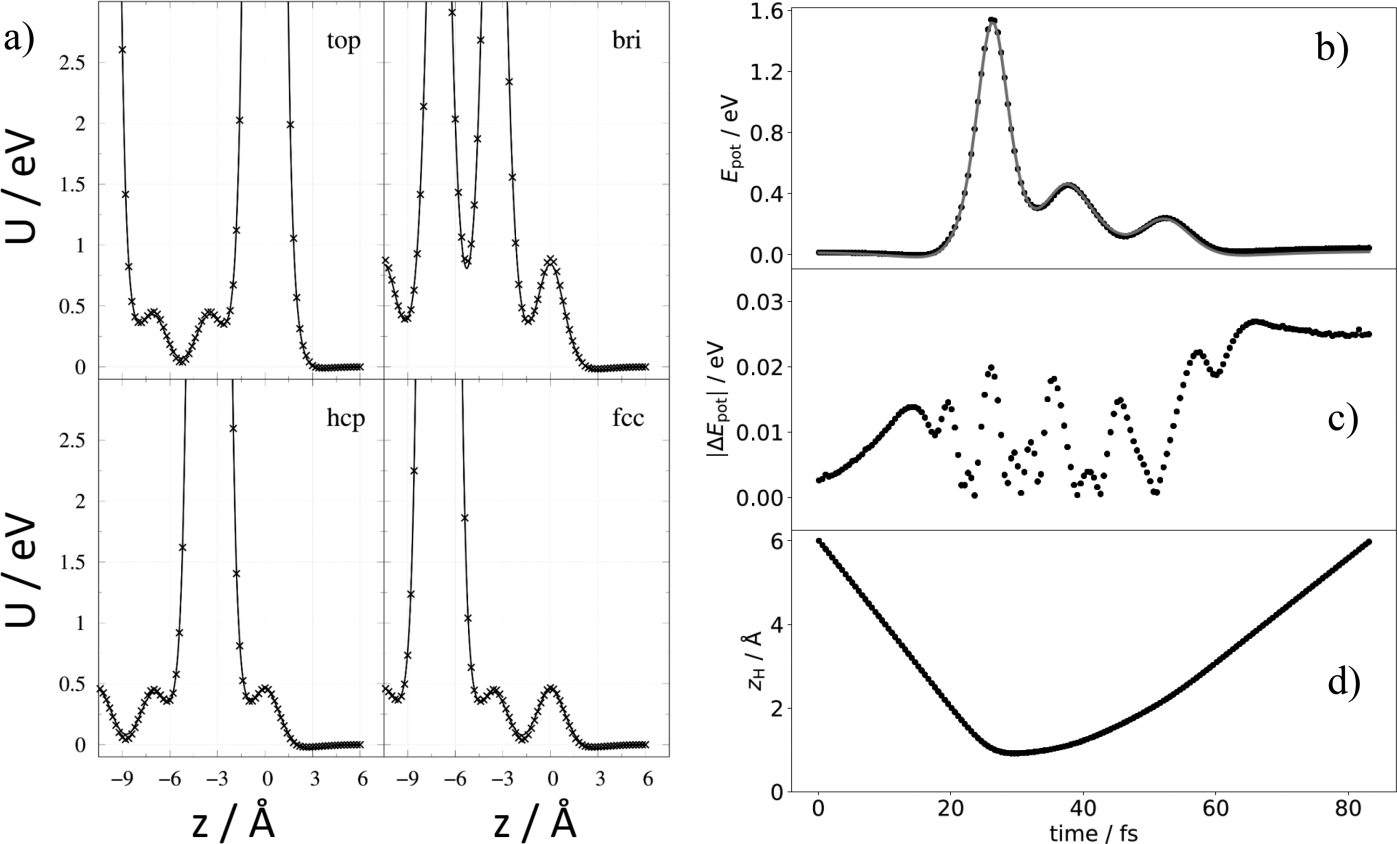
DFT data compared to the EMT function H interacting with solid Xe. (a) 1D cuts through the high-dimensional PES for an H atom moving along the surface normal at four different surface sites: top bridge, hcp hollow and fcc hollow. The DFT data is shown as “x” points, and the EMT fit is shown as solid lines. (b) Potential energy of the system for a scattering trajectory involving moving Xe atoms; here, DFT data (circles) are compared to EMT energies (solid line). (c) Energy differences between DFT and EMT are shown. (d) H atom distance to the surface during the trajectory. A coordinate system for H atom is employed, where *x* and *y* are parallel to the surface and *z* is along the surface normal. For a pictorial representation of the sites, see Figure 1 of ref (37)

The EMT function reproduces the DFT data for H on Xe with a RMSE of 0.024 eV; see Figure 1. This is the total energy deviation for our 17-atom system. The minimum energy structure represented by the PES corresponds to H atoms adsorbed at an fcc hollow where the H atom is 2.8 Å displaced toward the vacuum from the plane defined by the equilibrium positions of the first layer of Xe atoms. The binding energy is 0.03 eV. Subsurface interactions are also accurately described by the EMT-PES. Although not strictly comparable, a PES for H–Xe derived from molecular beam scattering experiments^34^ gives a similar H–Xe equilibrium distance and well depth. Figure 1c shows the deviation between DFT and EMT for a trajectory involving Xe atom motion. The excellent agreement is convincing evidence that the EMT PES accurately described the Xe–Xe interactions predicted by DFT.

**Figure 2:**
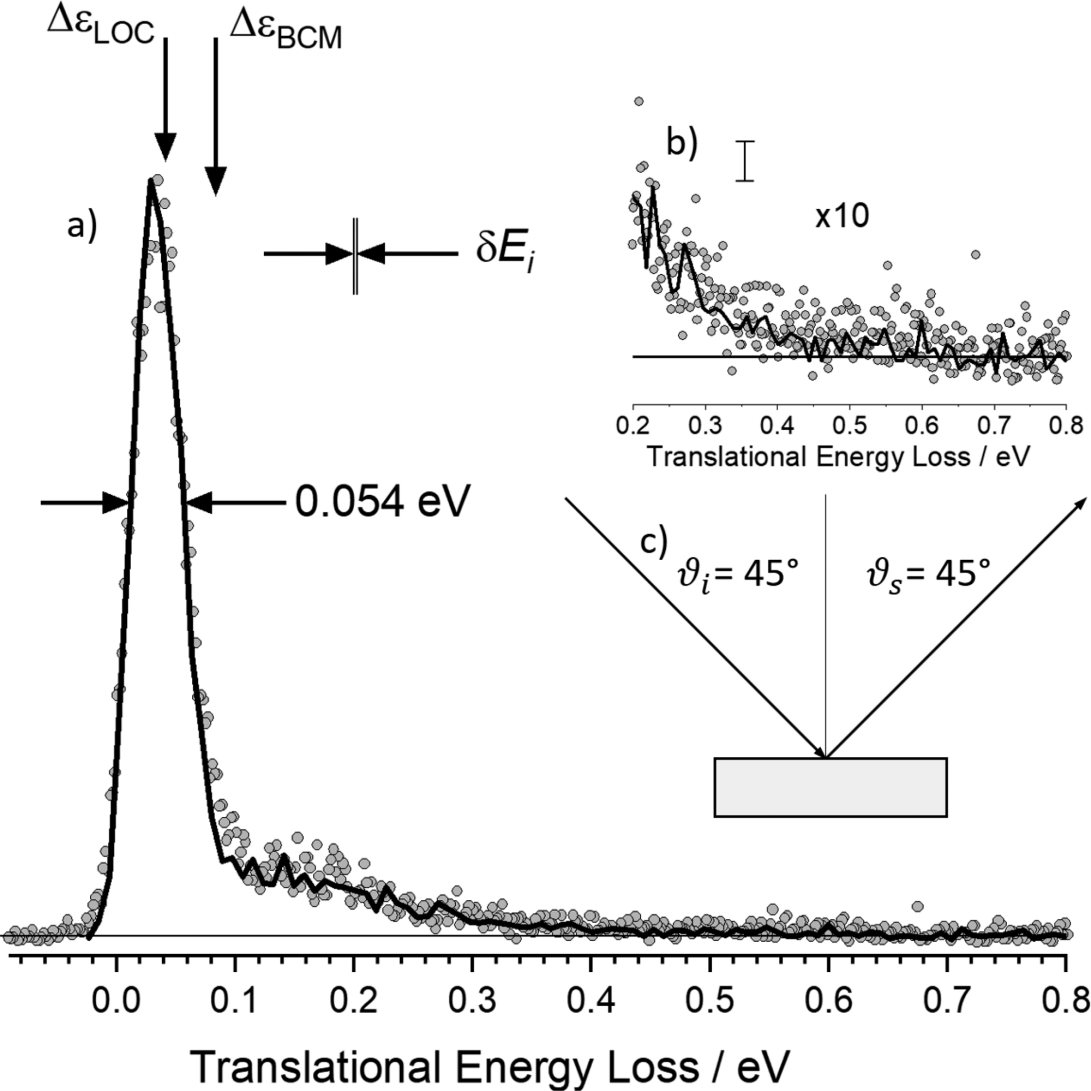
Energy-loss spectrum for H scattering from solid Xe. (a) Rydberg tagging experiment (circles) and MD simulation (solid line). The sharp peak dominating the energy distribution results from single-bounce LOC scattering and “weak double-bounce scattering”. The shoulder spanning 0.1–0.5 eV results from strong double-bounce and multibounce collisions including subsurface scattering. The inset in (b) shows a zoomed in view of the data with the largest inelasticity and an estimate of the statistical noise in the MD trajectories. (c) Experimental conditions: *E*_i_ = 2.76 eV, ϑ_i_ = 45°, ϑ_s_ = 45° and φ_i_ = 0°, and *T*_S_ = 45 K. The spread in the H atoms’ incidence energy δ*E*_i_ is also shown. The experimental data has been shifted to a lower energy loss by less than 0.015 eV, consistent with the experimental uncertainty in the absolute energy scale. The horizontal line shows the baseline level of the experiment. φ_i_ = 0° corresponds to the azimuthal orientation of the Xe surface where the projection of the velocity vector of the incident H atoms along the surface is parallel to the 110 direction of the crystal. In the MD simulations, the trajectories were culled, including only those within ±5° of the nominal scattering angle. This represents about 4000 trajectories of the total (1 million).

Figure 2 shows the energy-loss spectrum for H atoms scattering from solid Xe obtained with Rydberg atom tagging TOF (circles) and MD trajectory calculations (line). For both experiment and simulation, the H atom beam is incident 45° from the surface normal, and atoms are scattered at the specular angle. The spectrum comprises a dominant peak with an energy loss of 0.04 eV and a fwhm of 0.054 eV as well as a second feature with energy losses between 0.1 and 0.5 eV. The figure also shows the position of the energy loss predicted by a line-of-centers (LOC) model Δε_LOC_ = *E*_i_ cos^2^ ϑ_i_[1 – (*m*_Xe_ – *m*_H_)^2^/(*m*_Xe_ + *m*_H_)^2^], which is the fraction of the normal component of incidence energy lost to the surface while conserving momentum and assuming that H atom momentum parallel to the surface is unaffected by the collision. The expected energy loss for a binary collision Δε_BCM_ = *E*_i_[1 – (*m*_Xe_ – *m*_H_)^2^/(*m*_Xe_ + *m*_H_)^2^] is also shown.

The fact that the main peak in the energy-loss distribution is consistent with Δε_LOC_ is often taken as evidence for “single-bounce” dynamics. However, the LOC model obviously cannot explain the width of the observed energy-loss feature, nor can it explain energy losses greater than 0.04 eV. Furthermore, since Δε_BCM_ is the maximum amount of energy loss possible in a signal-bounce collision, multibounce collisions must play a role.

Because the MD simulations agree well with experiment, we have used them to investigate the scattering dynamics in this system in detail. This analysis reveals that both multibounce and subsurface direct inelastic scattering are important in H atom collisions at solid Xe under the experimental conditions of this work.

**Figure 3:**
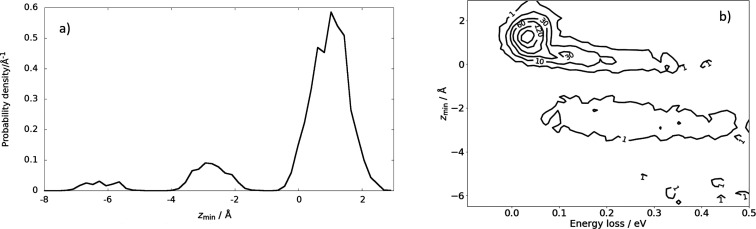
Subsurface scattering: (a) Probability density distribution of the scattering trajectories as a function of distance of closest approach to the surface *z*_min_. The equilibrium positions of the Xe surface atoms define *z*_min_ = 0. (b) Probability correlation distribution comparing depth of penetration and energy-loss. The numbers on the contour lines indicate the number of MD trajectories. *E*_i_*=* 2.76 eV, ϑ_i_ = 45°, ϑ_s_ = 45°, and φ_i_ = 0° and *T*_S_ = 45 K.

We first consider subsurface scattering. Figure 3a shows a histogram of the minimum values of the *z*-coordinate *z*_min_ found in the trajectories contributing to the energy-loss distribution of Figure 2. (In our coordinate system, *z* is the distance from the plane defined by the equilibrium positions of the surface Xe atoms.) The largest feature in this distribution peaks at 1 Å, corresponding to surface scattering without penetration, but a substantial fraction of the scattering events exhibit negative values of *z*_min_ with a peak at −3 and −6 Å. These trajectories travel deep within the Xe solid before re-entering the gas phase. Figure 3b shows how energy loss increases with depth of penetration, exhibiting energy losses that span the high energy-loss feature of the experimentally obtained distribution shown in Figure 2. Two representative trajectories traversing the first and second subsurface sites are shown as Movies S1 and S2, respectively. Inspection of the trajectories reveals that subsurface scattering involves many H–Xe collisions.

We next consider how one can count the number of H–Xe collisions (bounces) associated with each trajectory. To do this, we must first understand that the definition of a bounce is fundamentally ambiguous. Hence, the bounce number is only meaningful with knowledge of the definition. To appreciate this ambiguity, consider a collision between a high-energy gas-phase H atom and a stationary Xe atom. Technically, any interaction that results in a change in the H atom’s direction of travel, no matter how small, qualifies as a collision (bounce), despite the fact that collisions producing large deflection angles transfer much more energy than collisions with low deflection angles. In short, we need a way to classify collisions according to their ability to transfer energy between the H and Xe atoms.

**Figure 4:**
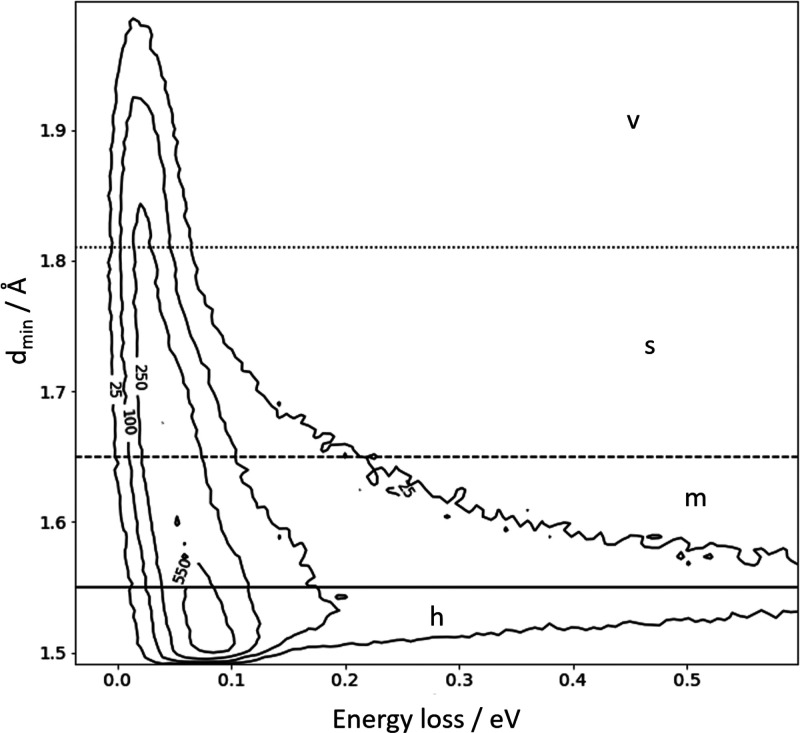
Two-dimensional histogram for the scattering-angle-integrated distance of closest approach of an H atom to a Xe atom in H scattering from solid Xe. *E*_i_ = 2.76 eV, ϑ_i_ = 45° and *T*_S_ = 45 K. We note the correlation between *d*_min_ and energy loss. The horizontal lines set boundaries between hard (h), medium (m), soft (s), and very soft (v) collisions. According to our classification, hard collisions are those where the H atom approaches a Xe atom within 1.55 Å achieving a potential energy of 2.46 eV. Medium collisions require a *d*_min_ of 1.65 Å and achieve 1.82 eV, while soft collisions approach 1.81 Å, producing an interaction energy of 1.1 eV at the turning point. The numbers on the contour lines indicate the number of MD trajectories.

To make progress, consider Figure 4, which shows the scattering-angle-integrated histogram of H–Xe distances of closest approach *d*_min_ for all the trajectories run in our MD simulation. All trajectories exhibit *d*_min_ values between about 1.5 and 2 Å. The figure shows a correlation of energy loss with *d*_min_. Obviously, collisions that approach more closely collide with smaller impact parameters and transfer more energy. It is therefore convenient to divide this distribution into four categories: hard (h), medium (m), soft (s), and very soft (v) collisions, according to the value of *d*_min_ as shown in Figure 4. With this definition in mind, we can begin to analyze the number of bounces in each trajectory.

**Figure 5:**
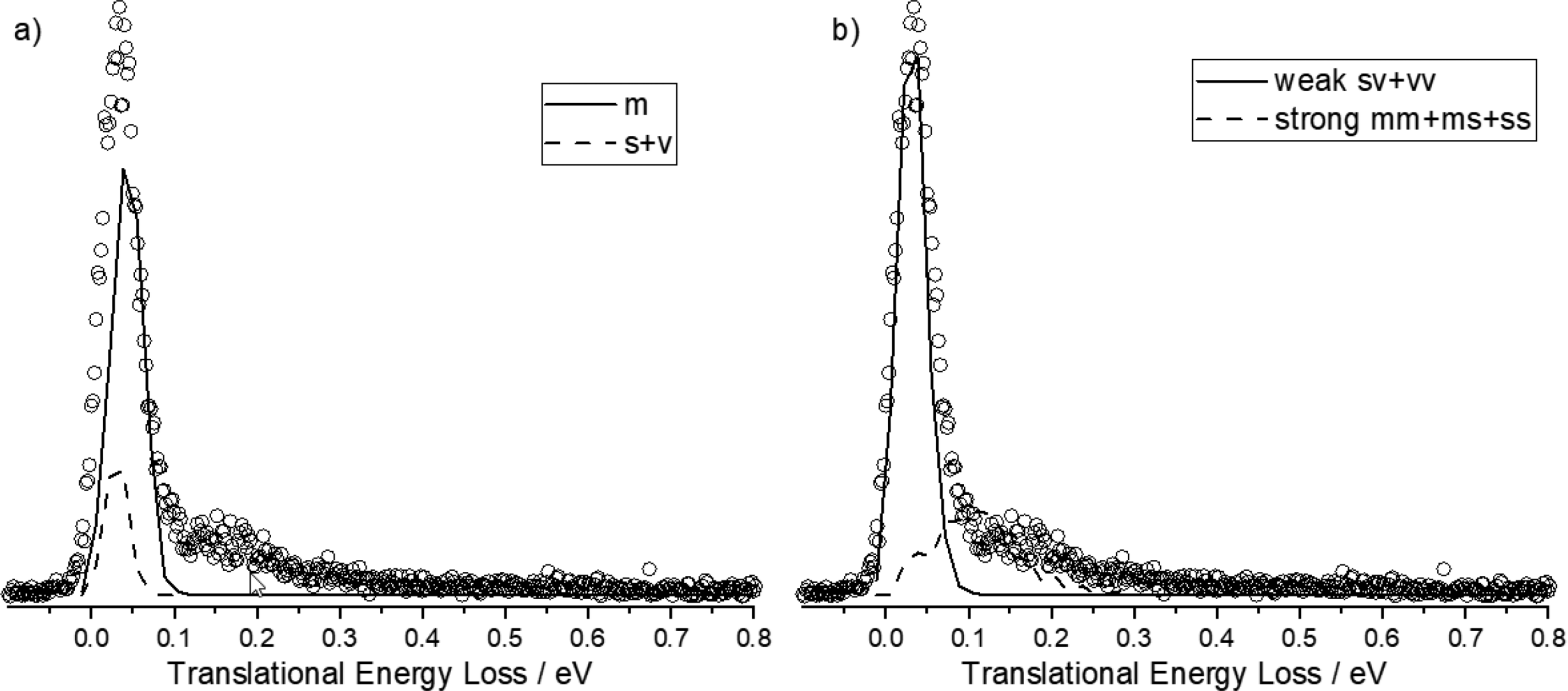
Decomposition of the energy-loss spectrum into single- and double-bounce trajectories. (a) Single-bounce trajectories are found only in the energy-loss feature near 0.04 eV. (b) Double-bounce trajectories are of two types. Weak double-bounce trajectories are found within the energy-loss feature at 0.04 eV and strong double collisions help explain the feature with energy loss greater than 0.1 eV. The unaccounted for scattering signal is due to higher numbers of bounces (not shown). The experimentally derived energy-loss distribution is shown for reference as open circles. The normalization of MD and experiment in each case is done to try to give the best agreement.

Figure 5a,b shows single and double-bounce trajectories that contribute to the energy-loss distribution shown in Figure 2. The dominant feature of the experimental distribution seen at about 0.04 eV arises partly from medium single-bounce trajectories. The remaining single-bounce trajectories belong to either the soft or very soft category and make up a small portion of the scattering signal. A typical medium single-bounce trajectory is shown in Movie S3. In Figure 5b, double-bounce trajectories are shown. Weak double-bounce trajectories (vv + sv) contribute to the low energy loss side of the peak centered at 0.04 eV. A typical sv double-bounce trajectory is shown in Movie S4.Without weak double-bounce trajectories, it is impossible to account for the full width of the main energy-loss feature seen in experiment. In fact, only a minority (47%) of all the trajectories scattered into all final angles are the result of single-bounce events. Figure 5b also shows that strong double-bounce (mm + ms + ss) trajectories account for most of the high energy-loss feature out to about 0.25 eV. A typical ms double-bounce trajectory is shown in Movie S5.

**Figure 6:**
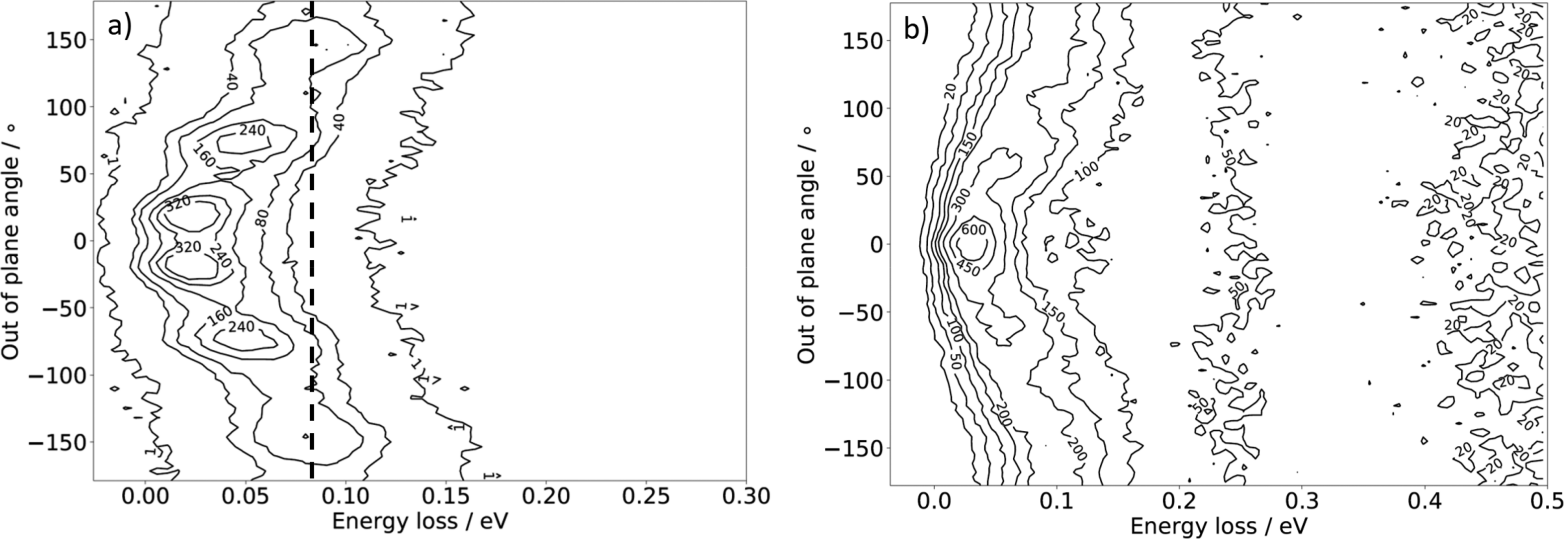
Out-of-plane scattering of H from solid Xe when integrated over all polar scattering angles. (a) Single-bounce trajectories. The vertical dashed line indicates the energy loss predicted by the binary collision model. (b) Multibounce scattering trajectories. The numbers on the contour lines indicate the number of MD trajectories. *E*_i_ = 2.76 eV, ϑ_i_ = 45°, and *T*_S_ = 45 K.

Hard single-bounce trajectories do not contribute to the experimental energy-loss distribution seen in Figure 2 as such collisions do not produce scattered H atoms in the plane of detection. Figure 6 shows the out-of-plane angle dependence of the scattered H atom flux integrated over all in-plane scattering angles. Single-bounce trajectories (Figure 6a) show a clear correlation between energy loss and out-of-plane scattering angle: The harder the collision, the larger the out-of-plane scattering angle. In fact, the hardest possible collisions at Δε_BCM_ (vertical dashed line in Figure 6a) only occur for out-of-plane scattering angles approaching π, which reflects backscattered H atoms traveling in the opposite direction of the incident beam.

Interestingly, single-bounce trajectories have a diminished probability at any energy loss to be scattered in the detection plane. In contrast, weak double-bounce trajectories (energy-loss ∼0.04 eV) peak within the detection plane; see Figure 6b. This surprising observation reflects the fact that pairs of out-of-plane scattering events can cancel the out-of-plane momentum; effectively, they are guided by collisions with Xe atoms on opposite sides of the detection plane (see Movies S4 and S5). Strong multibounce trajectories behave more as expected; they are scattered to all out-of-plane angles.

This analysis shows that without high-resolution angle-resolved inelastic scattering capability like that offered by H atom Rydberg tagging, observation of multibounce and subsurface scattering would be difficult if not impossible. The differential scattering experiments presented here are, however, able to resolve specific dynamical events in surface scattering. These diagrams also point out that the energy losses that will be seen in different laboratories will depend on the precise geometry of the experiment. For example, some scattering experiments relying on ion imaging collect a larger fraction of out-of-plane scattering than the present experiments. Indeed, many are done exclusively with ϑ_i_ ∼ 0°, meaning that the back-scattered BCM limit is more easily observed.

Before closing, we would like to mention a few observations relating to MD simulations carried out on a Lennard-Jones (LJ) pair potential. The LJ pair PES is often the method of first resort for constructing a PES, but we show here that such a simple approach can lead to serious qualitative problems in describing the interatomic interactions. In our case, the LJ-PES is extremely simple to construct, as parameters for LJ pair potentials for H/Xe and Xe/Xe are easily obtained;^34,35^ see Table 2. Using these parameters, we easily produced a full-dimensional PES and repeated some of the calculations we had carried out on our more expensive EMT-PES.

**Figure 7:**
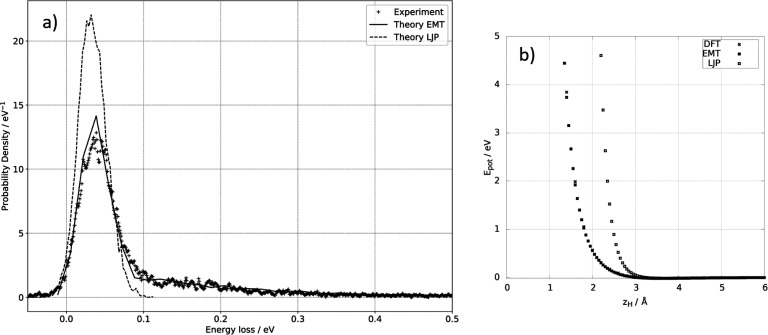
Performance of a Lennard-Jones pair potential. (a) Comparison with experiment of MD trajectory simulations using two potential energy surfaces. While the results using the EMT-PES are in excellent agreement with experiment, the LJ-PES fails to capture the high energy losses seen between 0.1 and 0.5 eV. (b) Comparison of the repulsive interaction of H with Xe for the EMT-PES and the LJ-PES. The EMT PES agrees well with DFT, whereas the LJ-PES gives the effective size of the Xe atom to be nearly 1 Å larger for an H atom colliding at 2.76 eV energy. This suppresses penetration and sticking.

Figure 7a shows the energy-loss distribution calculated with the LJ-PES compared to the results obtained with the EMT-PES and to experiment. With EMT-PES, the MD simulations are able to capture the experimental results extremely well. The results obtained with the LJ-PES are markedly worse. Nonetheless, the LJ-PES MD simulations also reproduce the main feature seen in experiment at 0.04 eV. In fact, one might consider the deviations acceptable for many applications, but such a conclusion could be dangerous.

The high energy losses between 0.1 and 0.5 eV, shown above to be due to multibounce and penetrating trajectories, are completely absent in the MD simulations resulting from the LJ-PES. We also computed the sticking probability using the LJ-PES to be about 5 × 10^–6^. Comparing this to 0.15 found when using the EMT-PES, one begins to have greater dissatisfaction with the performance of the LJ-PES.

Both of these deficiencies are a result of errors in the repulsive H–Xe interaction given by the LJ approximation. Figure 7b shows the comparison of the DFT, EMT, and LJ-PESs in a way that emphasizes the repulsive interaction between H and Xe at energies relevant to this work. This shows that the DFT and EMT PESs are substantially softer than is LJ. The effective radius of each Xe atom is nearly 1 Å larger under the LJ approximation at a collision energy of 2.76 eV, and this error in the effective size of the Xe atom persists to incidence energies well below 1 eV. It is for this reason that the sticking probability is markedly reduced as sticking requires penetration to the subsurface.

## 4 Conclusions

The scattering of H from solid Xe provides a special opportunity to delve into the dynamical details of atomic collisions at simple solid surfaces. The combination of high-resolution differential scattering experiments combined with high-dimensional dynamical simulations allows for this. In the course of this study, we find evidence that while conventional single-bounce dynamics reported frequently in the literature of surface scattering is clearly important, other dynamical scattering processes can also be identified that are just as or even more likely. Within the context of a definition of weak and strong collisions based on the distance of closest approach during the trajectory, we find that single-bounce trajectories cannot account for the full energy-loss distribution seen in experiment. In fact, only 47% of all trajectories are the result of single-bounce events. Double-bounce trajectories are more important even for specular scattering where one might think single-bounce events would be favored. The tendency of each bounce to direct H atoms out of the plane of detection allows two bounces to compensate out of plane momentum and more easily remain in the detection plane. These weak double-bounce events exhibit nearly the same energy loss as that predicted by single-bounce line of centers model. This may explain why they have not been experimentally resolved in the past. We also observe that a large fraction of the observed scattering results from trajectories that visit regions of space below the first layer of Xe atoms (subsurface multibounce scattering) before returning to the gas phase. Overall, these multibounce and subsurface scattering dynamics allow as much as 0.5 eV to be lost from 2.76 eV H atoms colliding with a solid Xe surface, far exceeding the predicted energy loss of the binary collision model (0.082 eV) normally considered the largest energy loss possible. Subsurface penetration is also responsible for sticking of the H atom, which we compute to occur for 15% of the trajectories. A LJ pair potential fails to describe penetration or sticking mainly due to its inability to accurately describe the repulsive wall of the H–Xe interaction.

## Supporting Information Available

Five movies showing example trajectories that illustrate the dynamics of H scattering from solid xenon: Movies S1 and S2: multibounce traversing the first and second subsurface sites (MP4 and MP4); Movie S3: medium single-bounce trajectory (MOV); Movie S4: soft/very soft double-bounce trajectory (MP4); Movie S5: medium/soft double-bounce trajectory (MP4)

## Supplementary Material

jp1c03433_si_002.mp4

jp1c03433_si_003.mov
